# Prognostic impact of fractional flow reserve measurements in patients with acute coronary syndromes: a subanalysis of the FLORIDA study

**DOI:** 10.1007/s00380-023-02256-7

**Published:** 2023-04-17

**Authors:** Teresa Gerhardt, Barbara E. Stähli, Tanja K. Rudolph, Matthias Lutz, Anne-Sophie Schatz, Lukas Zanders, Tino Schubert, Magnus Stueve, Nick E. J. West, Els Boone, Ulf Landmesser, David M. Leistner

**Affiliations:** 1 Klinik für Kardiologie, Charité Campus Benjamin Franklin, Universitätsmedizin Berlin, Hindenburgdamm 30, 12203 Berlin, Germany; 2 Klinik für Kardiologie, Universitäres Herzzentrum, Universitätsspital Zürich, Zurich, Switzerland; 3 Herz- und Diabeteszentrum Nordrhein-Westfalen, Universitätsklinik der Ruhr-Universität Bochum, Bad Oeynhausen, Germany; 4 Universitätsklinik Schleswig-Holstein, Kiel, Germany; 5 LinkCare GmbH, Ludwigsburg, Germany; 6 Abbott Vascular, Santa Clara, CA USA; 7 Berlin Institute of Health (BIH), 10117 Berlin, Germany; 8 DZHK (German Centre for Cardiovascular Research), Partner Site North, 24105 Kiel, Germany; 9 DZHK (German Centre for Cardiovascular Research), Partner Site Berlin, 12203 Berlin, Germany; 10 University Heart & Vascular Center Frankfurt, Frankfurt, Germany; 11 DZHK (German Centre for Cardiovascular Research), Partner Site Rhein/Main, Frankfurt, Germany

**Keywords:** Fractional flow reserve, Acute coronary syndrome, Unselected, Prognosis, Long-term follow-up

## Abstract

Randomized trials suggest benefits for fractional flow reserve (FFR)-guided vs. angiography-guided treatment strategies in well-defined and selected patient cohorts with acute coronary syndromes (ACS). The long-term prognostic value of FFR measurement in unselected all-comer ACS patients, however, remains unknown. This subanalysis of the Fractional FLOw Reserve In cardiovascular DiseAses (FLORIDA) study sought to investigate the long-term effects of FFR in the management of lesions in patients with acute coronary syndrome (ACS). FLORIDA was an observational all-comer cohort study performed in Germany, that was population-based and unselected. Patients enrolled into the anonymized InGef Research Database presenting with ACS and undergoing coronary angiography between January 2014 and December 2015 were included in the analysis. Patients were stratified into either the FFR-guided or the angiography-guided treatment arm, based on the treatment received. A matched cohort study design was used. The primary endpoint was all-cause mortality. The secondary endpoint was major adverse cardiovascular events (MACE), a composite of death, non-fatal myocardial infarction (MI), and repeat revascularization. Follow-up time was 3 years. Rates of 3-year mortality were 10.2 and 14.0% in the FFR-guided and the angiography-guided treatment arms (*p* = 0.04), corresponding to a 27% relative risk reduction for FFR in ACS patients. Rates of MACE were similar in both arms (47.7 vs. 51.5%, *p* = 0.14), including similar rates of non-fatal MI (27.7 vs. 25.4%, *p* = 0.47) and revascularization (9.9 vs. 12.1%, *p* = 0.17). In this large, all-comer observational study of ACS patients, FFR-guided revascularization was associated with a lower mortality at 3 years. This finding encourages the routine use of FFR to guide lesion revascularization in patients presenting with ACS.

**Supplementary Information:**

The online version contains supplementary material available at 10.1007/s00380-023-02256-7.

## Introduction

Patients presenting with acute coronary syndromes (ACS) are at high risk for recurrent major adverse cardiovascular events (MACE) [1]. Appropriate early recognition and revascularization of non-culprit coronary lesions that do induce myocardial ischemia, while avoiding risks associated with excess use of stents for lesions that have no physiological relevance is crucial to optimize outcome in this vulnerable patient group [2]. Especially in the context of acute myocardial infarction (MI), assessment of the functional relevance of non-culprit stenoses can be challenging and unreliable, among others due to hemodynamic disturbance and scattered thrombus material. Fractional flow reserve (FFR) is a well-established index used to assess the functional significance of coronary lesions with high specificity and high spatial resolution, that can be measured with a pressure-wire during coronary angiography [2, 3]. In patients with chronic coronary syndrome (CCS), various landmark clinical trials have shown that FFR measurement to guide judicious use of stents can improve outcome compared to percutaneous coronary intervention (PCI) guided solely by angiography or conservative treatment [2–6]. There is growing evidence of the role of FFR guidance for revascularization of non-culprit stenoses in the setting of ACS, but data are conflicting. Clinical data supporting FFR-guided management in the setting of ACS is either limited to subgroups of patients with ACS or based on the results of relatively small clinical trials, all in very selected patient collectives [7–11]. Additionally, in most studies, FFR-guided revascularization was compared to optimal medical therapy (OMT) alone, and direct comparisons with angiography-guided revascularization are lacking [7, 12, 13]. The Fractional FLOw Reserve In cardiovascular DiseAses (FLORIDA) study was a large observational cohort study, based on anonymized German health claims data. The goal of the present subgroup analysis was to extend the current knowledge on the value of FFR guidance to an all-comer ACS patient population. We additionally sought to investigate outcomes stratified by treatment received, namely revascularization by PCI or coronary artery bypass grafting (CABG) and optimal medical therapy (OMT).

## Methods

### Study design and data source

The FLORIDA study was an observational cohort study, based on the InGef (Institute for Applied Health Research Berlin, Berlin, Germany) database, an anonymized German health claims dataset. Eligible patients underwent at least one inpatient coronary angiogram for suspected coronary artery disease between January 2014 and December 2015. Patients eligible for this subgroup analysis presented either with ST-segment elevation myocardial infarction (STEMI), non-ST-segment elevation myocardial infarction (NSTEMI) or unstable angina (UA). Patients in the FFR-guided treatment arm underwent FFR assessment of coronary lesions either at the time of coronary angiography and PCI for the index ACS event or as a staged procedure during the index hospitalization.

A detailed nearest-neighbor matching design adjusting for 72 variables from propensity score estimation (see Supplemental Material for a list of covariates included in the propensity score estimation), as well as age, and sex was used to match 635 patients receiving FFR-guided treatment for lesions with 635 patients receiving angiography-guided treatment, selected from a total of 28,960 patients undergoing coronary angiography for ACS. Baseline characteristics, medical treatments, and comorbidities were determined using inpatient and outpatient health claims for the 365 days prior to the index ACS event. Patients were stratified by the type of treatment received, i.e., PCI, CABG, or conservative management with OMT alone, based on the procedure codes during the index hospitalization.

### Outcome definitions

The primary endpoint was all-cause mortality. The secondary endpoint was rates of MACE, a composite of mortality, non-fatal MI, and repeat revascularization at 3 years. Non-fatal MI was defined as urgent hospitalization and revascularization due to non-ST-elevation- or ST-elevation MI (NSTEMI, STEMI). Repeat revascularization was defined as any repeat PCI required during follow-up that was not related to an acute MI, including revascularization due to disease progression, restenosis or stent thrombosis. The analysis period ended either at the first hierarchized MACE or at the end of the 3-year follow-up period, whichever happened first.

### Statistical analysis

Continuous variables are presented as mean ± standard deviation (SD) or median (interquartile range) as appropriate, and categorical variables as numbers and percentages. The balance of the characteristics of the treatment arms was evaluated using standardized mean differences (SMD), defined as the absolute difference in means (or proportions) divided by the average SD. Differences in variables were assessed using Chi-squared tests with Yates’ correction for categorical variables and two-sample *t *tests for continuous variables.

Follow-up time for MACE was censored at the date of first MACE. For the hierarchized analysis of MACE components, follow-up was censored at death, first non-fatal MI for patients who survived the follow-up period, or first revascularization for patients who survived and did not suffer from a MI in the follow-up period. A log-rank test was used to compare hazard rates regarding MACE for the two treatment arms which were also presented as Kaplan–Meier survival curves. A 2-sided *p* value < 0.05 was considered to indicate statistical significance. All data were analyzed using SAS^®^ 9.4 (SAS Institute Inc., Cary, NC, USA) and R 3.4.0 (The R Foundation for Statistical Computing, Vienna, Austria).

## Results

### Baseline characteristics

**Fig. 1:**
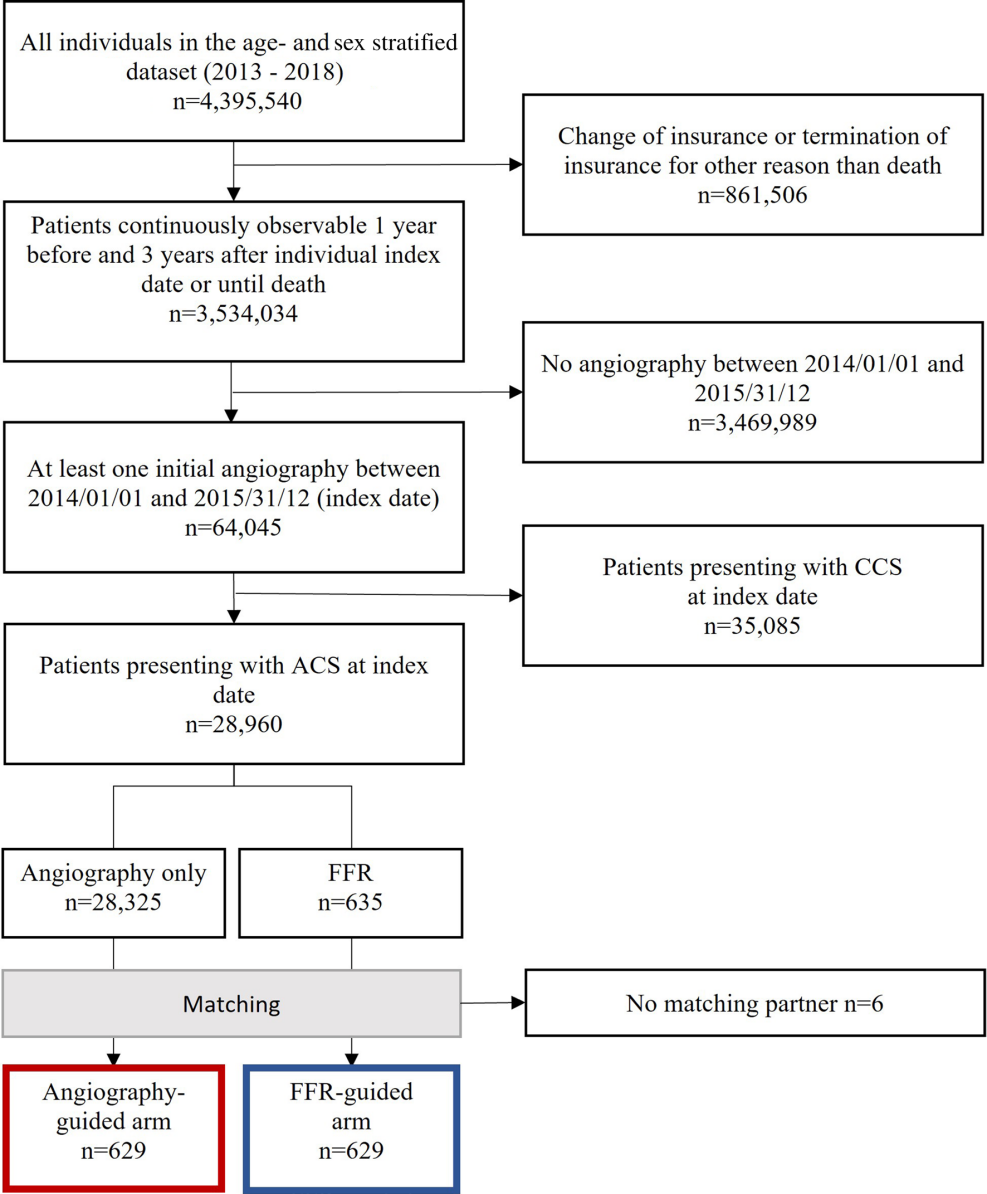
Flow diagram of the study. All patients presenting with ACS who received at least one angiography were included. Patients were matched for sex, age ± 5 years, and estimated propensity scores, with each patient in the FFR-guided treatment group matched to the closest patient in the angiography-guided treatment group. *ACS* acute coronary syndrome, *CCS* chronic coronary syndrome, *FFR* fractional flow reserve, *n* number

Out of 28,960 patients that presented with ACS in the FLORIDA study population, FFR was measured in 635 (2.7%) patients (Fig. 1). Table 1 shows baseline characteristics of all ACS patients in the study. Mean age of the patients was 68.3 ± 13.5 years and 15,974 patients (66.7%) were male. Patients in the FFR-guided treatment arm were slightly older (69.1 ± 11.4 vs. 68.3 ± 13.5 years, *p* = 0.14), more likely to have prior coronary artery disease (55.6 vs. 43.6%, *p* < 0.001), and were more often on beta blocker therapy (53.4 vs. 44.5%, *p* < 0.001) and statin therapy (49.3 vs. 38.0% *p* < 0.001). After matching for estimated propensity scores, age, and sex, both treatment arms consisted of 629 patients (Fig. 1 and Table 2). Of the 691 patients with ACS, 38.8% in the FFR arm and 40.4% in the non-FFR arm presented with STEMI or NSTEMI while the remaining patients presented with unstable angina pectoris (*p* = 0.604). A further stratification of myocardial infraction in STEMI and NSTEMI was not possible due to missing data availability.

**Table 1:** Baseline and procedural characteristics

	Total study cohort	FFR-guided treatment arm	Angiography-guided treatment arm	SMD in %
				(*p* value)
*N*	28,960	635	28,325	–
Demographics				
Male sex	15,974 (66.7)	432 (68.0)	15,542 (66.6)	2.98 (0.4869)
Mean age	68.3 (13.5)	69.1 (11.4)	68.3 (13.5)	5.85 (0.1392)
Comorbidities/risk factors				
Known CAD	10,526 (43.9)	353 (55.6)	10,173 (43.6)	24.13 (< 0.0001)
Known heart failure	4533 (18.9)	113 (17.8)	4,420 (18.9)	2.98 (0.4956)
Renal failure	340 (1.4)	11 (1.7)	329 (1.4)	2.59 (0.6126)
Diabetes mellitus	8859 (37.0)	222 (35.0)	8637 (37.0)	4.31 (0.3060)
Arterial hypertension	18,070 (75.4)	511 (80.5)	17,559 (75.3)	12.53 (0.0032)
Obesity	5101 (21.3)	143 (22.5)	4958 (21.3)	3.06 (0.4726)
Dyslipidemia	13930 (58.1)	418 (65.8)	13,512 (57.9)	16.31 (< 0.0001)
Medical therapy				
Antiplatelets	5587 (23.3)	184 (29.0)	5403 (23.2)	13.27 (0.0008)
Beta blockers	10,711 (44.7)	339 (53.4)	10,372 (44.5)	17.91 (< 0.0001)
ACE inhibitors/ARB	7743 (32.3)	216 (34.0)	7527 (32.3)	3.71 (0.1727)
Statins	9168 (38.3)	313 (49.3)	8855 (38.0)	22.99 (< 0.0001)

*ACE* angiotensin converting enzyme, *ARB* angiotensin receptor blocker, *CAD* coronary artery disease, *FFR* fractional flow reserve, *N* number, *SMD* standard mean difference

**Table 2:** Baseline and procedural characteristics after matching

	Total study cohort	FFR-guided treatment arm	Angiography-guided treatment arm	SMD in %
				(*p* value)
*N*	1258	629	629	–
Demographics				
Male	856 (68.0)	428 (68.0)	428 (68.0)	0 (> 0.9999)
Mean age	69.0 (11.4)	69.0 (11.4)	69.0 (11.4)	0 (> 0.9999)
Age < 65 years	430 (34.2)	215 (34.2)	215 (34.2)	0 (> 0.9999)
Age 65 ≤ 75 years	354 (28.1)	176 (28.0)	178 (28.3)	0.71 (0.9500)
Age ≥ 75 years	474 (37.7)	238 (37.8)	236 (37.5)	0.66 (0.9536)
Comorbidities/risk factors				
Known CAD	691 (54.9)	347 (55.2)	344 (54.7)	0.96 (0.9300)
Known heart failure	221 (17.6)	112 (17.8)	109 (17.3)	1.25 (0.8822)
Renal failure	20 (1.6)	11 (1.7)	9 (1.4)	2.54 (0.8217)
Diabetes mellitus	469 (37.3)	220 (35.0)	249 (39.6)	9.55 (0.1026)
Arterial hypertension	1015 (80.7)	505 (80.3)	510 (81.1)	2.01 (0.7751)
Obesity	280 (22.3)	142 (22.6)	138 (21.9)	1.53 (0.8389)
Dyslipidemia	824 (65.5)	414 (65.8)	410 (65.2)	1.34 (0.8588)
Medical therapy				
Antiplatelets	374 (29.7)	179 (28.5)	195 (31.0)	5.57 (0.3548)
Beta blockers	670 (53.3)	336 (53.4)	334 (53.1)	0.64 (0.9549)
ACE inhibitors/ARB	434 (34.5)	213 (33.9)	221 (35.1)	2.68 (0.6780)
Statins	627 (49.8)	309 (49.1)	318 (50.6)	2.86 (0.6519)

*ACE* angiotensin converting enzyme, *ARB* angiotensin receptor blocker, *CAD* coronary artery disease, *FFR* fractional flow reserve, *SMD* standard mean difference

Coronary revascularization was performed in 362 (57.5%) and 380 (60.4%) patients in the FFR-guided and the angiography-guided treatment arms, respectively. There was no significant difference in the number of stents received during index hospitalization between groups: the mean number of stents was in the index hospital stay: mean number of stents was 1.48 (FFR) and 1.60 (non-FFR), respectively (*p* = 0.1758).

In the FFR-guided treatment arm, 358 (56.9%) patients underwent PCI and 4 (0.6%) patients CABG. In the angiography-guided treatment arm, 374 (59.5%) patients underwent PCI and 6 (0.9%) patients CABG. A total of 267 (42.3%) and 249 (39.6%) patients in the FFR-guided and the angiography-guided treatment arms received OMT only (*p* = 0.3445).

### Mortality

**Fig. 2:**
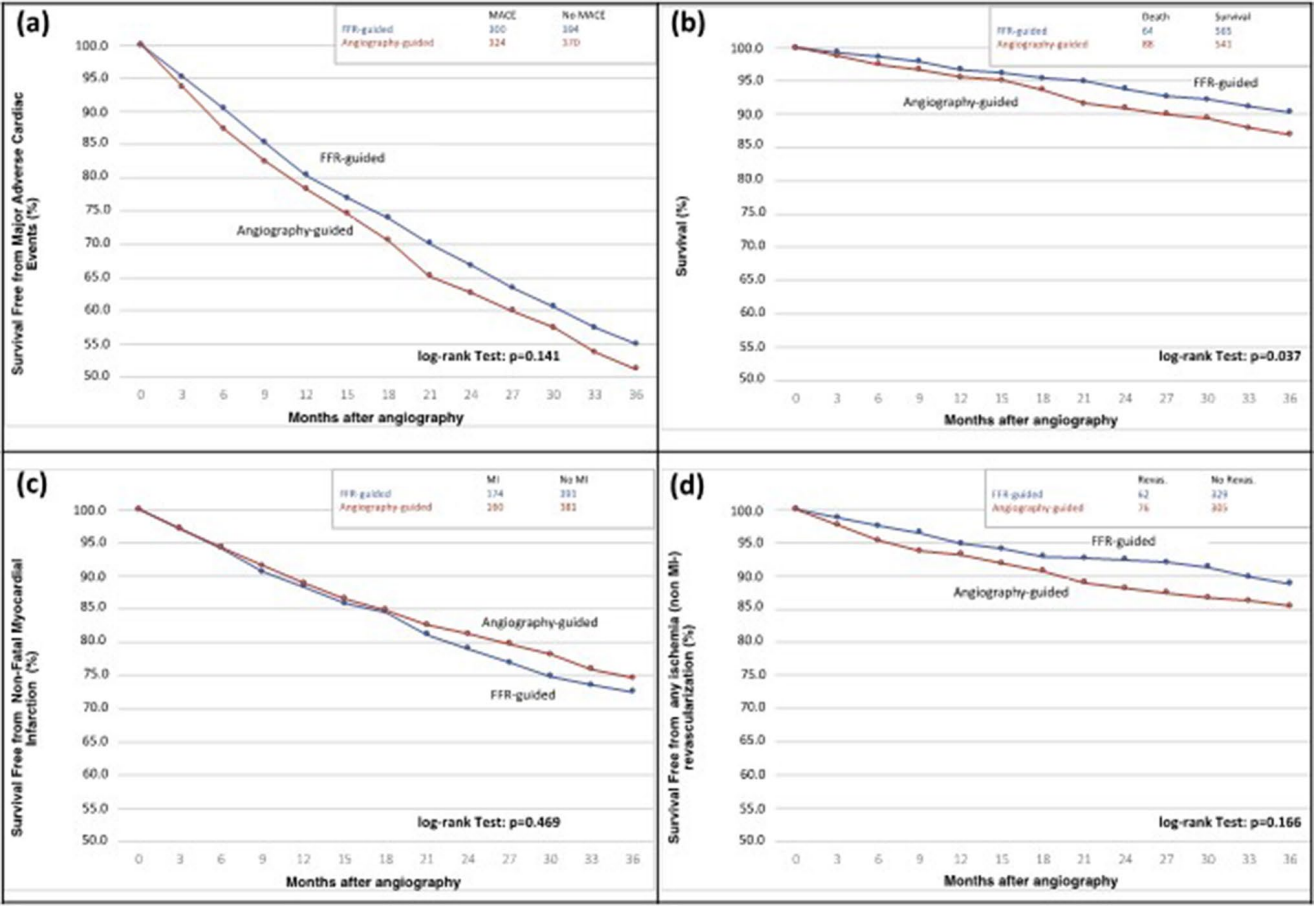
MACE in ACS patients with FFR-guided vs. angiography-only management. **a** MACE-free survival, **b** survival, **c** survival free from non-fatal MI, and **d** survival free from non-MI-associated revascularization. *FFR* fractional flow reserve, *MACE* major adverse cardiovascular events, *MI* myocardial infarction

**Fig. 3:**
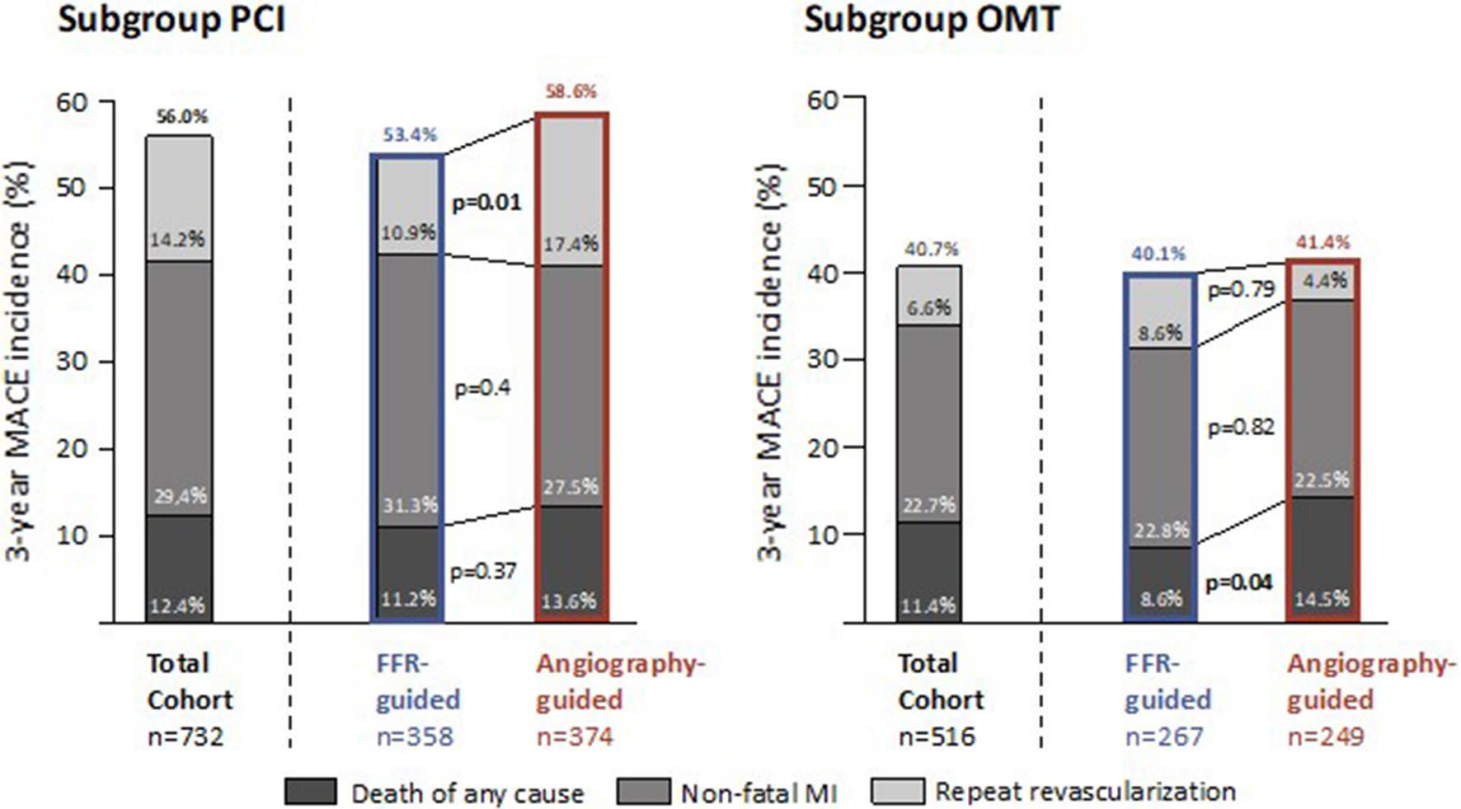
Rates of MACE at 3 years in pre-defined patient subgroups. *FFR* fractional flow reserve, *MACE* major adverse cardiovascular events, *MI* myocardial infarction, *PCI* percutaneous coronary intervention, *OMT* optimal medical therapy

Rates of mortality were 10.2% (64 patients) and 14.5% (88 patients) in the FFR-guided and the angiography-guided treatment arms (*p* = 0.04) after 3 years, corresponding to a 27% relative risk reduction (RRR) and a number needed to treat (NNT) of 27 to prevent one death with FFR guidance (Fig. 2). Rates of mortality at 1 and 2 years were 3.8% (24 patients) vs. 4.9% (31 patients) and 7.3% (46 patients) vs. 10% (63 patients) in the FFR-guided and the angiography-guided treatment arms, respectively. When PCI was deferred during index hospitalization (OMT only group), the rate of mortality was significantly lower in the FFR-guided as compared to the angiography-guided treatment arm (8.6 vs. 14.5%, *p* = 0.04), corresponding to a RRR of 40% and a NNT of 17 (Fig. 3). Among patients who underwent non-culprit lesion PCI during the index hospitalization, rates of mortality were similar between the FFR-guided and the angiography-guided treatment arm (11.2 vs. 13.6%, *p* = 0.37).

### Major cardiac adverse events

The rate of MACE at 3 years was 49.6% (624 patients). Rates of MACE at 1 and 2 years were 24.2% (305 patients) and 38.3% (482 patients), respectively. Rates of MACE were 47.7% and 51.5% in the FFR-guided and the angiography-guided treatment arms (*p* = 0.14; Fig. 2). Rates of non-fatal MI (27.7 vs. 25.4%, *p* = 0.47) and repeat revascularization (9.9 vs. 12.1%, *p* = 0.17) did not differ between the FFR-guided and the angiography-guided treatment arms (Table 3).

**Table 3:** MACE rates and components within the study cohort over 3-years of follow-up

	Mortality	MACE	Non-fatal MI	Repeat revascularization
Total study cohort (*n* = 1258)				
1 y	55 (4.4)	305 (24.2)	169 (13.4)	81 (6.4)
2 y	109 (8.7)	482 (38.3)	261 (20.7)	112 (8.9)
3 y	152 (12.1)	624 (49.6)	334 (26.6)	138 (11.0)
FFR-guided treatment arm (*n* = 629)				
1 y	24 (3.8)	145 (23.1)	87 (13.8)	34 (5.4)
2 y	46 (7.3)	230 (36.6)	140 (22.3)	47 (7.5)
3 y	64 (10.2)	300 (47.7)	174 (27.7)	62 (9.9)
Angiography-guided treatment arm (*n* = 629)				
1 y	31 (4.9)	160 (25.4)	82 (13.0)	47 (7.5)
2 y	63 (10.0)	252 (40.1)	121 (19.2)	71 (11.3)
3 y	88 (14.5)	324 (51.5)	160 (25.4)	76 (12.1)

*MACE* major adverse cardiovascular events, *MI* myocardial infarction, *n* number, *y* year

In the OMT group, rates of MACE were 40.1 and 41.4% in the FFR-guided and the angiography-guided treatment groups (*p* = 0.79). There were no differences in rates of non-fatal MI (22.8 vs. 22.5%, *p* = 0.82) and repeat revascularization (8.6 vs.4.4%, *p* = 0.79, Fig. 3). Among patients who received PCI during the index hospitalization, rates of MACE at 3 years were 53.4 and 58.6%, in the FFR-guided and the angiography-guided treatment arms (*p* = 0.16). Rates of non-fatal MI at 3 years were similar among groups (31.3 vs. 27.5%, *p* = 0.40). The rate of repeat revascularization was lower in the FFR-guided as compared to the angiography-guided treatment arm (10.9 vs. 17.4%, *p* = 0.01), corresponding to a RRR of 37% and a NNT of 16 (Fig. 3).

## Discussion

To the best of our knowledge, this is the first study to provide long-term data on FFR-guided vs. angiography-guided concepts for revascularization in a large, nationwide, unselected cohort of ACS patients. We present two major findings. First, long-term rates of mortality were significantly lower in ACS patients undergoing FFR-guided revascularization of non-culprit lesions. Second, mortality benefits in patients undergoing FFR-guided revascularization for non-culprit lesions were observed in the OMT only, but not in the revascularization group.

### FFR-guided revascularization in ACS patients

Especially in the acute setting of MI, the functional relevance of coronary lesions is often under- or over-estimated [2]. Fractional flow reserve guidance provides a tailored approach to determine if coronary revascularization is warranted or if stenting can be deferred due to lack of functional relevance. While superiority of an FFR-based deferral strategy has been proven in CCS patients, deferral in the context of ACS is not well studied until today [14, 15]. It is noteworthy that among 28,960 patients with ACS undergoing coronary angiography in the FLORIDA study, FFR was used in only a small fraction of 2.7% (635) of patients. In line with this finding, a US database study reported a similar proportion of ACS patients receiving FFR-guided revascularization [16]. This points to insecurities among interventionalists as to the use of FFR in the context of ACS and underlines the need for more robust data to guide clinical decision making.

### FFR-guided revascularization and outcomes in ACS patients

The value of FFR to guide revascularization strategies beyond the initial management of the culprit lesion in the context of ACS remains controversial [9]. In the complete revascularization vs. treatment of the culprit lesion only in patients with ST-segment elevation myocardial infarction and multivessel disease (DANAMI-3 PRIMULTI) trial, a total of 627 patients presenting with STEMI and multivessel disease were randomized to either FFR-guided revascularization of non-culprit lesions or OMT only. Complete revascularization guided by FFR significantly reduced the risk of future cardiovascular events, largely driven by lower rates of repeat revascularization procedures [12, 13]. Likewise, in the Fractional Flow Reserve-Guided Multivessel Angioplasty in Myocardial Infarction (COMPARE-ACUTE) trial, a total of 885 patients with STEMI and multivessel disease were randomized to FFR-guided revascularization of non-culprit lesions or OMT only. Fractional flow reserve-guided revascularization resulted in a significantly lower rate of death from any cause, non-fatal myocardial infarction, revascularization, and cerebrovascular events [7, 17]. From these studies, however, no firm conclusions can be drawn regarding the comparison of FFR-guided vs. angiography-guided revascularization for non-culprit lesions in ACS patients, since FFR-based strategies were compared only to OMT, not to angiography-guided strategies. A post hoc analysis of the Fractional Flow Reserve vs. Angiography for Multivessel Evaluation (FAME) study demonstrated a similar reduction of adverse events with FFR guidance in both patients with NSTEMI or unstable angina and those with chronic coronary syndromes [8]. While this demonstrates feasibility of FFR not only in the stable, but also in the acute setting, it does not allow any conclusions as to superiority of FFR or angiography-only-based strategies in the acute setting. The fractional flow reserve vs. angiography in guiding management to optimize outcomes in non-ST-segment elevation myocardial infarction (FAMOUS-NSTEMI) trial randomized a total of 350 patients presenting with NSTEMI into a FFR-guided and an angiography-guided treatment arm for remaining non-culprit stenoses. Fractional flow reserve-guided management of ACS patients was associated with lower rates of coronary revascularization and higher rates of OMT compared to angiography-guided management [11]. This study, however, was restricted to patients with NSTEMI, follow-up was only 12 months and the rather small number of patients enrolled in the study precluded to draw firm conclusions on clinical outcomes among groups.

The recent Flow Evaluation to Guide Revascularization in Multivessel ST-Elevation Myocardial Infarction (FLOWER-MI) trial compared rates of death, MI, or urgent revascularization in STEMI patients with multivessel disease who received complete revascularization by an FFR-guided compared to an angiography-guided strategy. 1.5% in the FFR-guided group and in 1.7% in the angiography-guided group died; 3.1 and 1.7% suffered from non-fatal myocardial infarction; and unplanned hospitalization leading to urgent revascularization occured in 2.6 and 1.9%, respectively [18]. FFR-guided revascularization strategies were not associated with better outcomes. In the present observational study, with higher real-world rates of these MACE events, FFR showed its benefit explicitly in the OMT arm. It might be worth investigating potential benefits for MI patients without the need of PCI in randomized-controlled trials.

A large retrospective analysis from the US-National Readmissions Data (NRD) reported lower rates of in-hospital mortality for patients presenting with ACS and undergoing FFR guidance for PCI. Out of 304,548 patients discharged with a diagnosis of ACS and treated invasively within the index hospitalization, a total of 7,832 patients underwent FFR-guided invasive treatment. This was associated with significantly lower in-hospital all-cause mortality, but no follow-up data were provided from this study [16]. In the present cohort of all-comer ACS patients, FFR guidance was associated with a significantly lower long-term mortality as compared with angiography guidance, with a relative risk reduction of 27%. Hence, these results confirm previous data on the superiority of FFR-guided PCI over angiography-only approaches and substantially extends previous in-hospital findings to long-term outcomes in a real-world, all-comer cohort of ACS patients [11, 16].

Rates of non-fatal MI were 13.4% at 1 year and 26.6% at 3 years. These rates are rather high in comparison to previously reported data. In the FAMOUS-NSTEMI, FLOWER-MI, and DANAMI-3-PRIMULTI trials, 1-year rate of non-fatal MI was 7.4, 3.1, and 5%, respectively [11, 13, 18]. In the 3-year evaluation of COMPARE-ACUTE, 8.3% of the patients had suffered from recurrent myocardial infarction [17]. The higher rate of non-fatal MI observed in FLORDIA might be due to the unselected all-comer study population with a mean age of 69 years and to the fact that the majority of patients were at high risk. Given the retrospective design of FLORIDA, we cannot exclude that FFR was preferentially measured in a frailer and sicker patient population with more advanced coronary artery disease and a higher overall risk as compared to the highly selected patient populations included in randomized trials. The increased rates of recurrent MI observed in patients treated with PCI could indicate a higher baseline risk in these patients. Further, differences in the definition of MI may come into play and hamper direct comparisons among studies.

Rates of repeat revascularization were 5.4 vs. 7.5% at 1 year and 9.9 vs. 12.1% at 3 years for the FFR-guided and the angiography-guided arm, respectively. The differences between the groups could be due to the fact that FFR during the index hospitalization can help assess and treat high risk lesion that would otherwise require repeat revascularization within 3 years follow up [12, 13].

These rates are lower than the ones reported in other studies. In FAMOUS-NSTEMI, rates of repeat revascularization were 21% in the angiography-guided and 13.2% in the FFR-guided treatment arm [11]. Some of this observed difference may be due to the hierarchical MACE assessment performed in the present analysis: of all patients included in this analysis, a total of 281 (22.3%) patients underwent revascularization within 3 years, but 51% of those were MI-related and thus counted into a different group. Additionally, differences between studies may be due at least in part to the variable definitions of repeat revascularization used.

## Study limitations

Some limitations merit consideration. First, information on angiographic lesion severity, particularly with respect to the lesions interrogated with FFR, as well as on single or multivessel disease was not available in the database. Second, the identification of patients with unstable angina, NSTEMI, and STEMI was precluded. Third, the distinction between FFR measurements performed during the index procedure and those performed during staged procedures during the index hospitalization was not possible. This may have introduced some heterogeneity that we were unable to account for. However, effects of immediate and staged procedures may have affected both treatment groups, i.e., FFR-guided and angiography-guided PCI, similarly. Fourth, we were unable to differentiate between cardiac and non-cardiac death as information on the cause of death was not collected in the database. Fifth, we cannot exclude completely that some elective staged procedures may have been misclassified as events of repeated revascularization. However, we would only expect to see those in a very short term, likely not affecting long-term effects that were investigated in this study. Sixth, although we carefully adjusted the two arms with a combination of direct and a propensity score matching including 72 variables, our dataset did not comprise information on educational level, socio-demographic status, and lifestyle (e.g., smoking, and physical activity). Our study could not control for a range of factors not available in the database which may have swayed the physicians’ decision at the time of the procedure with respect to the use of FFR. It can, therefore, not be ruled out that frailer and sicker patients with complex anatomy were preferentially treated with angiography guidance due to reservation about their ability to tolerate FFR measurements, inability to advance pressure wire or concerns about overall prognosis, which may have led to an inclination toward medical therapy regardless of ischemia. Lastly, as the database represents a health claims database, the possibility of misclassification and miscoding of data cannot be ruled out completely and adjudication of events was not possible. However, as structured insurance data were used, any selection or reporting bias can be excluded.

## Conclusion

In this large, real-world all-comer observational study of ACS patients, an FFR-based revascularization strategy was associated with a 27% RRR of mortality at 3 years, without increasing rates of non-fatal MI or repeat revascularization. These findings could support the routine use of FFR to guide revascularization of lesions in patients presenting with ACS. However, further research is warranted to better understand in which subgroups of ACS patients, i.e., unstable angina, NSTEMI, and STEMI, FFR-guided revascularization strategies are most useful.

## Supplementary Information

Below is the link to the electronic supplementary material.

Supplementary file1 (DOCX 37 KB)
