# Supplementary material for: Prognostic impact of fractional flow reserve measurements in patients with acute coronary syndromes: a subanalysis of the FLORIDA study
Source: Heart Vessels. 2023 Apr 17;38(8):1009–18. doi: 10.1007/s00380-023-02256-7 (PMC10290038; doi:10.1007/s00380-023-02256-7)
Supplement: Supplementary file 1 — Supplementary file1 (DOCX 37 KB) [file 380_2023_2256_MOESM1_ESM.docx]

**Table S1: Variables used for propensity score estimations**

|  | **ATC/ICD/ICPM/ EBM-Code** | **Odds Ratio**  **(95%-Confidence Interval)** | **p-value** |
| --- | --- | --- | --- |
| **Medication** | | | |
| *Beta blocking agents* | | | |
| Beta blocking agents, plain, selective | C07AB | **1.176 (1.061 – 1.303)** | **0.0021** |
| Beta blocking agents, others | C07AA, C07AG, C07BA, C07BB, C07BG, C07CA, C07CB, C07CG, C07DA, C07DB, C07EA, C07EB, C07FB, C07FX | 0.93 (0.586 **–** 1.474) | 0.7563 |
| *Calcium antagonists* |  |  |  |
| Dihydropyridine derivatives | C08CA | 1.008 (0.784 **–** 1.297) | 0.9484 |
| Calcium antagonists, others | C08CX, C08DA, C08DB, C08EA,  C08EX , C08GA | 1.008 (0.791 **–** 1.286) | 0.9457 |
| *ACE inhibitor+ARB* |  |  |  |
| ACE inhibitors, plain | C09AA | 0.867 (0.697 **–** 1.078) | 0.1995 |
| ACE inhibitors and diuretics | C09BA | 0.987 (0.855 **–** 1.138) | 0.8539 |
| Angiotensin II antagonists, plain | C09CA | 1.077 (0.946 **–** 1.226) | 0.2639 |
| Angiotensin II antagonists and diuretics | C09DA | 1.013 (0.867 **–** 1.183) | 0.8722 |
| ACE inhibitor+ARB, others | C09BB, C09BX, C09DB, C09DX | 1.093 (0.886 **–** 1.349) | 0.4078 |
| *Statins* |  |  |  |
| HMG COA reductase inhibitors | C10AA | 1.135 (0.891 **–** 1.447) | 0.3046 |
| Statins, others | C10BA, C10BX | 1.156 (0.902 **–** 1.483) | 0.2521 |
| *Anti-platelet therapy* |  |  |  |
| Platelet aggregation inhibitors excluding heparin | B01AC | 0.826 (0.671 **–** 1.017) | 0.0724 |
| Thrombin inhibitors, others | B01AE, B01AF, B01AX | **1.32 (1.069 – 1.630)** | **0.0099** |
| Nitrates | C01DA, C01DX, C01EB18 | 1.069 (0.832 **–** 1.373) | 0.6004 |
| Aspirin | N02BA01, A01AD05, B01AC06 | 0.871 (0.526 **–** 1.442) | 0.5919 |
| Clopidogrel | B01AC04, B01AC34 | 0.924 (0.773 **–** 1.104) | 0.3834 |
| Ticagrelor and prasugrel | B01AC24, B01AC22 | 0.854 (0.600 **–** 1.215) | 0.3797 |
| Vitamin K antagonists plus NOACS | B01AA, B01AF02, B01AF01, B01AF03, B01AE07 | **0.815 (0.688 – 0.966)** | **0.0181** |
| Isosorbide mononitrate | C01DA14, C01DA58 | 0.794 (0.554 **–** 1.138) | 0.2092 |
| Fibrates | C10AB | 1.03 (0.702 **–** 1.513) | 0.8785 |
| *Other common comedication* |  |  |  |
| Alpha-adrenoreceptor antagonists | G04CA | 1.089 (0.919 **–** 1.29) | 0.3241 |
| Glucocorticoids | H02AB | **0.828 (0.715 – 0.959)** | **0.0117** |
| Thyroid hormones | H03AA | 0.992 (0.880 **–** 1.119) | 0.8968 |
| Fluoroquinolones | J01MA | 1.018 (0.889 **–** 1.166) | 0.7966 |
| Acetic acid derivatives and related substances | M01AB | 0.898 (0.789 **–** 1.022) | 0.1044 |
| Propionic acid derivatives | M01AE | 1.036 (0.932 **–** 1.151) | 0.5174 |
| Preparations inhibiting uric acid production | M04AA | 0.973 (0.848 **–** 1.116) | 0.6953 |
| Pyrazolones | N02BB | 1.02 (0.911 **–** 1.142) | 0.7277 |
| Proton pump inhibitors | A02BC | **1.118 (1.014 – 1.234)** | **0.0258** |
| Biguanides | A10BA | 1.085 (0.929 **–** 1.268) | 0.3006 |
| Sulfonamides, plain | C03BA | **0.783 (0.685 – 0.895)** | **0.0003** |
| **Diseases** |  |  |  |
| *Cardio-vascular diseases* |  |  |  |
| Chronic ischemic heart disease | I25 | **1.38 (1.227 – 1.553)** | **<0.0001** |
| Previous myocardial infarction - existing | I25.2 | 0.989 (0.864 **–** 1.133) | 0.8730 |
| Heart failure – NYHA I or unknown | I50.11, I50.9 | 0.842 (0.674 **–** 1.051) | 0.1282 |
| Heart failure – NYHA II or III | I50.12, I50.13 | **0.800 (0.651 – 0.983)** | **0.0333** |
| Heart failure – NYHA IV | I50.14 | 0.869 (0.650 **–** 1.163) | 0.3460 |
| Other cardiac arrhythmias | I49 | 1.038 (0.923 **–** 1.167) | 0.5351 |
| Previous stroke - acute | I21, I22 | 0.842 (0.695 **–** 1.020) | 0.0787 |
| Previous stroke - existing | I69 | 1.137 (0.900 **–** 1.438) | 0.2819 |
| Presence of cardiac and vascular implants and grafts | Z95 | **0.803 (0.707 – 0.911)** | **0.0007** |
| *Diabetes* |  |  |  |
| Type 2 diabetes mellitus | E11 | **0.699 (0.577 – 0.847)** | **0.0003** |
| Other diabetes mellitus | E10, E13, E14 | 1.19 (0.980 **–** 1.446) | 0.0797 |
| *Other cardio-vascular risk factors* |  |  |  |
| Essential (primary) hypertension | I11 | 1.022 (0.781 **–** 1.338) | 0.8747 |
| Disorders of lipoprotein metabolism and other lipidemias | E78.1 – E78.9 | 1.069 (0.889 **–** 1.285) | 0.4775 |
| Kidney failure | N19 | 1.127 (0.823 **–** 1.542) | 0.4557 |
| Hypercholesterolemia | E78.0 | 0.95 (0.787 **–** 1.147) | 0.5932 |
| Pulmonary Hypertension | I27 | 1.209 (0.904 **–** 1.618) | 0.2010 |
| Overweight and obesity | E66 | **1.36 (1.031 – 1.794)** | **0.0294** |
| *Other diseases* |  |  |  |
| Spondylosis | M47 | **1.155 (1.033 – 1.291)** | **0.0112** |
| Thoracic, thoracolum, and lumbosacral intvrt disc disorders | M51 | 1.069 (0.947 **–** 1.206) | 0.2801 |
| Dorsalgia | M54 | 1.011 (0.915 **–** 1.117) | 0.8338 |
| Hyperplasia of prostate | N40 | **1.159 (1.019 – 1.318)** | **0.0248** |
| Encounter for screening for malignant neoplasms | Z12 | 1.038 (0.935 **–** 1.152) | 0.4841 |
| Disorders of refraction and accommodation | H52 | 1.06 (0.960 **–** 1.170) | 0.2504 |
| Osteoarthritis of knee | M17 | **0.861 (0.764 – 0.969)** | **0.0135** |
| Presence of other functional implants | Z96 | 1.017 (0.904 **–** 1.145) | 0.7756 |
| **Previous interventions** | | | |
| Creation of an aortocoronary bypass | 5-361 | **0.121 (0.030 – 0.488)** | **0.0030** |
| Creation of an aortocoronary bypass by minimally invasive technique (Inpatient) | 5-362 | 0.325 (0.045 **–** 2.372) | 0.2680 |
| Inpatient angiocardiography | 1-276.0 | 0.438 (0.107 **–** 1.787) | 0.2501 |
| Other diagnostic catheter examination of the heart and vessels (inpatient) | 1-279 | **2.43 (1.573 – 3.754)** | **<0.0001** |
| Other outpatient intervention (PTCA, Stent) | 34286 | **0.281 (0.088 – 0.900)** | **0.0326** |
| Outpatient serial angiogram | 34284, 34283 | 1.18 (0.601 **–** 2.316) | 0.6308 |
| Placement of a drug-eluting bifurcation stent (inpatient) | 8-837.v | 0.22 (0.030 **–** 1.608) | 0.1356 |
| Transarterial left heart catheter examination (inpatient) | 1-275 | **1.31 (1.065 – 1.611)** | **0.0105** |
| Transarterial left heart catheter examination (outpatient) | 34291 | **1.728 (1.235 – 2.417)** | **0.0014** |
| *Previous stents in a coronary artery* | 8-837.k, 8-837.m |  |  |
| 1 stent |  | 1.228 (0.881 **–** 1.710) | 0.2254 |
| 2 stents |  | 0.856 (0.552 **–** 1.327) | 0.4859 |
| 2 stents in several coronary arteries |  | 0.751 (0.337 **–** 1.675) | 0.4846 |
| 3 stents |  | 1.317 (0.746 **–** 2.324) | 0.3421 |
| 3 stents in several coronary arteries |  | 0.946 (0.370 **–** 2.416) | 0.9077 |
| 4 stents |  | 0.739 (0.225 **–** 2.422) | 0.6173 |
| 4 stents in several coronary arteries |  | 0.419 (0.057 **–** 3.113) | 0.3956 |

Statistically significant predictors to α=0.05 are marked bold. Disease names correspond to the translation of ICD-10-GM codes, medication names to the description of the ATC classification and procedures to the translation of the German ICPM and EBM codes.

Abbreviations: EBM means Einheitlicher Bewertungsmaßstab, *Doctors' fee scale,* ICD-10-GM = International statistical classification of diseases and related health problems, 10^th^ revision, German modification, and ICPM = International classification of procedures in medicine
